# Developmental stochasticity and variation in floral phyllotaxis

**DOI:** 10.1007/s10265-021-01283-7

**Published:** 2021-04-05

**Authors:** Miho S. Kitazawa

**Affiliations:** Center for Education in Liberal Arts and Sciences, Osaka University, 1-16 Machikaneyama-cho, Toyonaka, Osaka 560-0043 Japan

**Keywords:** Evo-Devo, Floral development, Floral phyllotaxis, Stochasticity, Variation

## Abstract

Floral phyllotaxis is a relatively robust phenotype; trimerous and pentamerous arrangements are widely observed in monocots and core eudicots. Conversely, it also shows variability in some angiosperm clades such as ‘ANA’ grade (Amborellales, Nymphaeales, and Austrobaileyales), magnoliids, and Ranunculales. Regardless of the phylogenetic relationship, however, phyllotactic pattern formation appears to be a common process. What are the causes of the variability in floral phyllotaxis and how has the variation of floral phyllotaxis contributed to floral diversity? In this review, I summarize recent progress in studies on two related fields to develop answers to these questions. First, it is known that molecular and cellular stochasticity are inevitably found in biological systems, including plant development. Organisms deal with molecular stochasticity in several ways, such as dampening noise through gene networks or maintaining function through cellular redundancy. Recent studies on molecular and cellular stochasticity suggest that stochasticity is not always detrimental to plants and that it is also essential in development. Second, studies on vegetative and inflorescence phyllotaxis have shown that plants often exhibit variability and flexibility in phenotypes. Three types of phyllotaxis variations are observed, namely, fluctuation around the mean, transition between regular patterns, and a transient irregular organ arrangement called permutation. Computer models have demonstrated that stochasticity in the phyllotactic pattern formation plays a role in pattern transitions and irregularities. Variations are also found in the number and positioning of floral organs, although it is not known whether such variations provide any functional advantages. Two ways of diversification may be involved in angiosperm floral evolution: precise regulation of organ position and identity that leads to further specialization of organs and organ redundancy that leads to flexibility in floral phyllotaxis.

## Introduction

**Fig. 1:**
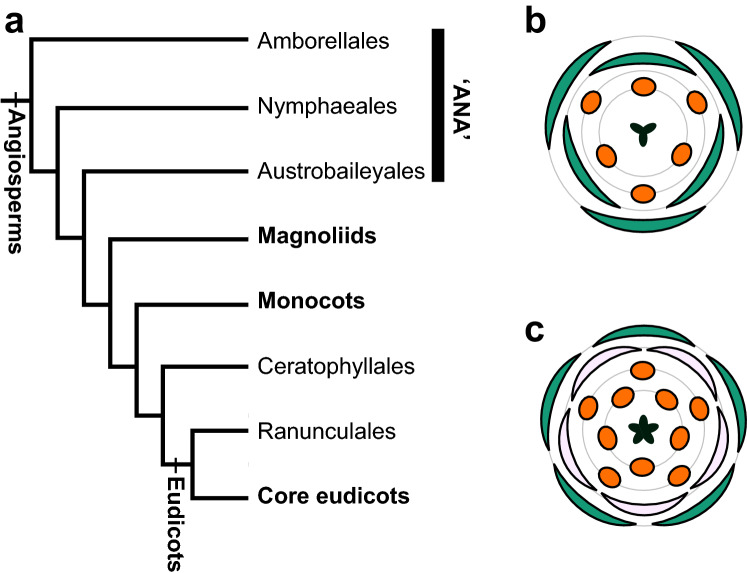
Angiosperm phylogeny and floral ground plans. **a** Simplified angiosperms phylogeny (modified from Angiosperm Phylogeny Group 2016). Some branches are not presented since they are not mentioned in this paper. **b** Monocot and **c** eudicot floral ground plans, depicted by floral diagrams, which are widely used to discuss floral diversity (Ronse De Craene 2010)

Besides the remarkable diversity in form of angiosperm flowers, floral organ numbers and arrangements are relatively invariant, especially in core eudicots and monocots (Fig. 1). Floral phyllotaxis, the arrangement of floral organs, is largely classified into whorled (cyclic) and spiral. In contrast to core eudicots and monocots, which usually show whorled phyllotaxis, different angiosperm clades show wide floral phyllotaxis variation within and among species. Solving the evolutionary history of floral phyllotaxis is not a simple problem, since both spiral and whorled phyllotaxis are observed in taxa in ‘ANA’ grade (Fig. 1a) that are sister to the major diversification of angiosperms (Smyth 2018). Recently, the ancestral floral phyllotaxis was reconstructed as whorled in the perianth and the androecium, and spiral in the gynoecium (Sauquet et al. 2017). This reconstructed ancestral flower has triggered arguments on the evolution of floral phyllotaxis, especially regarding its development (De-Paula et al. 2018; Rümpler and Theißen 2019; Sokoloff et al. 2018). The development of phyllotaxis has been studied over a century, under both experimental and theoretical studies. Application of these theories to floral phyllotaxis will benefit studies on floral evolution.

Recently, studies focusing on phenotypic variation and developmental stochasticity have advanced rapidly, including the research fields related to phyllotaxis. Traditionally, the average, usually the mean but sometimes the mode, is considered, and variations are often ignored. Mean-centric approaches, however, lose a lot of information (Geiler-Samerotte et al. 2013). We have begun to understand that organisms can utilize stochasticity in various processes, such as organ development and cell-fate decisions (Meyer and Roeder 2014). As sessile organisms, plants have the ability to respond to fluctuating environments by changing their development, which results in varied and flexible architectures and phenotypes. For some traits, variations could be a result of adaptive evolution (Abley et al. 2016). In this review, I provide an overview of the molecular and cellular stochasticity in the developmental processes and instability of phyllotaxis, which is helpful in understanding floral organ positioning and how stochasticity contributes to floral development and evolution.

## Stochasticity in plant development

Biological systems ubiquitously exhibit stochasticity in traits and processes, from the molecular to the multicellular level, even in developmental processes of robust and consistent phenotypes. Computational and experimental approaches have been used to study stochasticity in biological systems (Meyer and Roeder 2014). For example, a recent review on stochasticity in cellular fate decisions compared it to a weighted coin toss (Zechner et al. 2020). We have now begun to understand the mechanisms underlying the figurative coin toss model and to identify the factors contributing to the associated coin weights in various developmental processes.

### Robust development of multicellular tissue under molecular, cellular, and environmental stochasticity

Cells in identical genetic and environmental backgrounds can show phenotypic diversity. It has been shown that gene expression shows stochasticity caused by intrinsic noise (fluctuations), produced by biochemical processes involved in gene expression, and extrinsic noise from other cellular components (Elowitz et al. 2002; Kærn et al. 2005; Raser and O’Shea 2005). In multicellular organisms, stochasticity in gene expression can be affected by multicellularity. For example, the stochasticity of the protein expression level among neighboring cells in young leaves is spatially correlated (Araújo et al. 2017). The correlation is not sufficiently explained by the inheritance of mRNA and proteins, suggesting that the inheritance of cellular condition and/or cell–cell communication is required (Araújo et al. 2017).

Variability in gene expression is not the only source of phenotypic variations. Examples from animals have shown that mechanical properties of cells and molecular fluctuations in the dynamics of signaling pathways affect cellular fate decision (Kroll et al. 2020; Weinreb et al. 2020; Zechner et al. 2020). Significant advancements have been made in research on stochasticity in cellular size and patterning (Meyer and Roeder 2014). Cellular sizes fluctuate around the mean established by the ploidy (Roeder et al. 2010), and the dynamics of endoreduplication that cause ploidy variation are identified as a stochastic Poisson process (Kawade and Tsukaya 2017). Spatial variation in the growth rate among neighboring cells is several folds in magnitude and sub-cellular differences in cell periphery have been observed (Elsner et al. 2012). Heterogeneity in turgor pressure correlating with cellular topology and geometry is observed in shoot apexes, although it is noted that heterogeneity may not always be stochastic (Long et al. 2020). Cell division orientation can be stochastic, indicated by a population of similarly shaped cells showing different division planes, contrary to the traditional idea that the division plane is uniquely determined by cellular shape (Besson and Dumais 2011). Moreover, plant aerial parts are exposed to fluctuating environments, and have response mechanisms to buffer these fluctuations (Walter and Schurr 2005). Such stochasticity in various aspects of cellular properties and environmental noises can affect multicellular development.

Several ways to control the noise, including molecular, cellular, or environmental noise, have been suggested, such as regulating the frequency of transcription and translation, controlling gene copy number, regulation by network motifs such as negative feedback, network redundancy, intercellular signaling, physical constraint, and mechanical feedback (Abley et al. 2016; Lachowiec et al. 2016; Lempe et al. 2013; Raj and van Oudenaarden 2008; Raser and O’Shea 2005). Local rules that describe interactions between components of systems, such as the connection of genes in gene regulatory networks or cell–cell communication in multicellular tissues, are often sufficient to form robust patterns (Long and Boudaoud 2019). Additionally, cellular or morphological redundancy can contribute to maintaining function where stochasticity in processes is affected. In vulval development of the nematode *Caenorhabditis elegans*, there are greater numbers of precursor cells than are actually required for functional vulvae, and only half of them adopt a vulval fate. Although the position of the cells adopting a vulval fate vary slightly among individuals, most of the non-canonical phenotypes are functional (Braendle and Félix 2008). This suggests that cellular redundancy plays a buffering role. Similarly, plants have morphological redundancy at a larger scale, such as repetitive organ (e.g., leaf) formation and reticulated vein networks that allow multiple alternative pathways for transportation of water and photosynthetic products (Lachowiec et al. 2016). Such redundancy can maintain functional robustness under fluctuations.

### Utilizing stochasticity during plant development

Contrary to traditional views that considered variable phenotypes to be the result of poorly buffered development, recent studies suggest that it may be favored in some ecological scenarios, such as fluctuating and unpredictable environments (Abley et al. 2016; Kærn et al. 2005; Vogt 2015). Stochasticity is an important component prevalent in biological systems, including plant development, and stochastic developmental variation contributes to phenotypic diversity. Stochasticity can also play a constructive role in the development and cellular differentiation of multicellular organisms. Plants have systems that enhance cellular heterogeneity in growth (Uyttewaal et al. 2012) and it has been suggested that heterogeneity is required to produce specific tissue mechanical properties such as sepal curvature (Roeder et al. 2010, 2012) and stable organ shape (Hong et al. 2016). Stochasticity can be used to promote robustness through spatiotemporal averaging to maintain diversity in cell size in leaf epidermises and/or to create subtle differences among cells that initiate patterns by breaking symmetry (Long and Boudaoud 2019; Meyer and Roeder 2014; Roeder 2018). Stochasticity can cause phenotype shifts associated with network architecture such as the re-wiring of key-gene connections in response to changing environments (Lachowiec et al. 2016) and positive feedbacks that can result in switching between multiple states (Raj and van Oudenaarden 2008). The effect of noise can appear not only at the cellular level, but also at the tissue or organ level. For example, noise in the pattern formation process can alter the resulting organ position (Mirabet et al. 2012). These facts suggest that stochasticity plays an important role in plant development.

## Instability in phyllotaxis

**Fig. 2:**
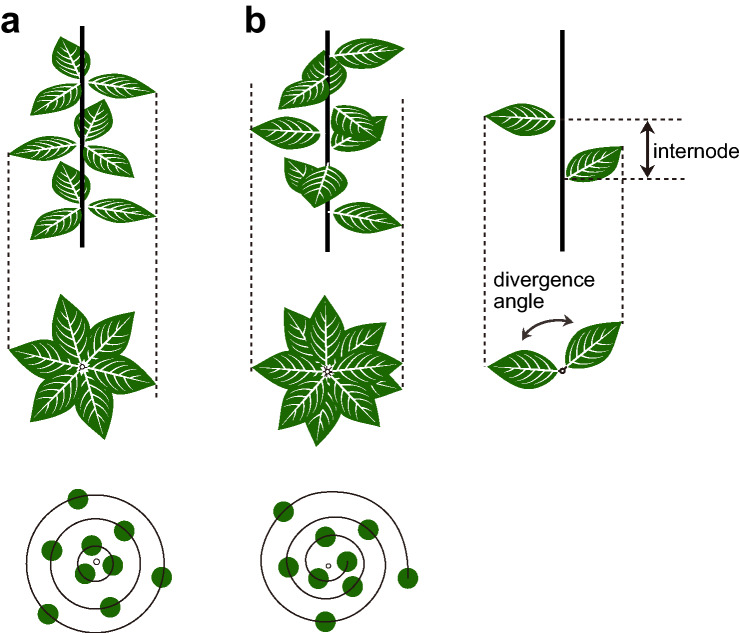
Major phyllotactic patterns. **a** Whorled and **b** spiral phyllotaxis. In each column, the upper panel is the lateral view and the middle panel is the top view. In the bottom panels, positions of lateral organs are indicated by solid circles. Black lines in **a** indicate the whorls, whereas in **b** the black line indicates an ontogenic spiral

Phyllotaxis is the arrangement of repeated units, represented by leaves, around a plant stem (Fig. 2). Plants fall mostly into two categories: spiral phyllotaxis, where the divergence angle between successive two organs is constant, and whorled phyllotaxis, in which several organs occur at the same level on the stem. Both classes differ in their development. In spiral phyllotaxis, organ primordia arise sequentially in constant plastochrons (time-intervals) and constant divergence angles. Whereas in whorled phyllotaxis, several primordia comprising a whorl initiate simultaneously and equidistantly from the shoot apex. Spiral phyllotaxis with a divergence angle of approximately 137.5° (the golden angle) is widely observed in the plant kingdom, from bryophytes to angiosperms, and even in distantly related taxa such as brown algae (Peaucelle and Couder, 2016).

The regularity of phyllotactic patterns has attracted the attention of many researchers over the centuries, with systematic studies beginning in the 1830s (Adler et al. 1997). Advantages of the common phyllotactic patterns in light capture and package efficiency have been discussed and several developmental mechanisms to achieve regularity have been suggested; for example, physical contact pressure among leaf primordia and chemical inhibitory fields. In angiosperms, lateral organ primordia are successively initiated at the shoot apex; therefore, phyllotactic patterns form around the shoot apical meristem (SAM). The central zone (CZ) of a SAM is an undifferentiated stem cell niche, and the peripheral zone (PZ) is the region where the cells undergo differentiation and where organ primordia are initiated. Theoretical studies have suggested the importance of SAM size on phyllotactic patterns. In *Arabidopsis thaliana* (L.) Heynh., CLAVATA-WUSCHEL (CLV-WUS) feedback signaling has been suggested as a central regulatory pathway for maintenance of the stem cell population in SAM (Brand et al. 2000; Schoof et al. 2000), and this regulatory pathway is likely conserved among angiosperms (Somssich et al. 2016). Feedback loop regulation enables the robust maintenance of the meristem size, even when the expression level of *CLV3* varies over a ten-fold range (Müller et al. 2006). Organ primordia initiation occurs in the PZ at the maxima of plant hormone auxin concentration. Auxin is transported by the efflux carrier PIN1 upward to the apex and accumulates, specifying the position of the primordium during early stages (Reinhardt et al. 2003). The expression of auxin carriers suggests that the heterogeneous distribution at the surface layer is the main requirement for organ pattern formation. Computer models have shown that simple local mechanisms, where a difference in auxin concentration among the cells causes the polarization of auxin efflux carriers, are sufficient to generate common phyllotactic patterns, such as the golden spiral and decussate pattern (Jönsson et al. 2006; Smith et al. 2006).

### Variation in phyllotaxis: fluctuations, transition, and permutation

Although it has been emphasized that the golden spiral and other regular patterns are widely observed in nature and many theoretical models succeeded to explain its stability, the observed occurrences of phyllotactic patterns are not invariant. Variation in phyllotaxis is often found in many species. There are roughly three types of variations: fluctuation around average, transition between multiple modes, and stochastic changes of the sequence, known as permutation.

Fluctuation around the average value has been observed in multiple species and mutants. For example, observations of mature *Rhododendron* branches showed that although the mean divergence angle is usually well-defined, individual divergence angles showed variations in branches (Bursill and Rouse 1998). In *A. thaliana*, divergence angles vary around the golden angle in wildtypes, and the degree of variation increases in some mutants. The variation in divergence angles and initiation timing can be generated around SAM; for example, stochasticity can exist in auxin dynamics or SAM and/or organ primordia size. In a quadruple mutant of auxin influx carriers, the divergent angle became irregular and PIN1 polar localization displayed a more diffuse pattern compared to the wild type, suggesting that rapid intake of auxin by influx carriers is required for stable regular patterning (Bainbridge et al. 2008). Mutants of genes regulating methyl esterification status of cell wall pectins show different degrees of variation in divergence angles around SAM compared to wildtypes, which can either be higher or lower depending on the mutated gene, suggesting that cell wall properties at primordium formation control the variability of primordium position (Peaucelle et al. 2011). Post-meristematic modification can also cause variations in the divergence angles and internode lengths on mature stem. In post-meristematic development, several genes, represented by *CUP-SHAPED COTYLEDON* (*CUC*) genes, are required to establish the boundary domain surrounding organ primordia. In plants expressing an *miR164*-resistant *CUC2* gene with a broader boundary domain, phyllotactic patterns are disrupted with a uniform distribution of divergence angles, in contrast to the maximum at 120°–150° in wildtype plants (Peaucelle et al. 2007). This irregularity is observed only in mature patterns and not in organ initiation patterns around SAMs (Burian et al. 2015; Peaucelle et al. 2007). The degree of variation is also affected by environmental conditions, such as day-length, associated with changes in SAM size (Landrein et al. 2015). Interestingly, the degree of variation is not the lowest in wildtypes in *A. thaliana* (Landrein et al. 2015; Peaucelle et al. 2011), suggesting a possibility that plants regulate it to keep a specific degree of variation.

In the theoretical models, multiple phyllotactic patterns including the golden spiral are generated and the transition between patterns occurs by changing phyllotactic parameters, such as size ratio between primordium and meristem. In nature, transitions between regular phyllotactic patterns are often observed. Transition between decussate to spiral phyllotaxis is commonly found in ontogeny of dicots, as they develop from two oppositely positioned cotyledons (Couder 1998; Meicenheimer 1998). Transitions can occur not only between spiral patterns but also between spiral and whorled phyllotaxis in the ontogeny where the area of the apical dome increases with time (Kwiatkowska and Florek-Marwitz 1999). Transitions are rather common when the developmental phase is shifted, for example from the vegetative to the reproductive phase, but it occurs even in the same phase generating primordia with the same identity (Kwiatkowska 1997; Kwiatkowska and Florek-Marwitz 1999). In the transient pattern between two regular patterns, different patterns or anomalous forms can appear, including oscillations in divergence angles (Smith et al. 2006) and an incompletely split leaf in the increased number of organs within a whorl in whorled phyllotaxis (Douady and Couder 1996c). The transitions are affected by how phyllotactic parameters change. For instance, types of transition changes depending on whether SAM size changes in a global and symmetrical manner or only in localized sectors (Meicenheimer and Zagorska-Marek 1989; Zagórska-Marek 1987). In mutants of model plants, non-random changes of phyllotactic patterns are observed. In maize, the mutant with the *ABERRANT PHYLLOTAXY1* (*ABPH1*) gene, which encodes cytokinin response regulator, shows opposite (decussate) phyllotaxis with enlarged SAM, in contrast to the distichous phyllotaxis in wildtypes (Giulini et al. 2004; Jackson and Hake 1999). In *A. thaliana*, three members of PLETHORA (PLT)-like AP2 domain transcription factors redundantly control organ positioning. The triple mutant of *PLT* genes shows nonrandom change in phyllotaxis, where successive leaves can either be at 180° or 90° (Prasad et al. 2011). Although it is suggested that PLT proteins act as PIN1 regulators, the alternation of phyllotaxis in *plt* mutant is not directly caused by PIN1 level but by the accumulation of auxin at the center of SAM (Pinon et al. 2013). These studies suggest that meristem size as well as auxin dynamics play a central role in the transition between regular phyllotactic patterns.

Irregular sequences of organ arrangement or permutation are observed in many species (Barabé and Lacroix 2020; Refahi et al. 2016). A major example of permutation is an “M-shaped” motif appearing in spiral phyllotaxis of a divergence angle α°, showing a transient sequence of 2α°, 360–α°, 2α° (Besnard et al. 2014; Couder 1998; Guédon et al. 2013). This motif is observed even in *A. thaliana* wildtypes, and appeared much more frequently in a mutant of cytokinin signaling inhibitor AHP6 (Besnard et al. 2014). This mutant shows a regular divergence angle and irregular plastochrons, and it is suggested that cytokinin regulates organ initiation after positioning by auxin, parallel with or downstream of auxin (Besnard et al. 2014). Studies on a mutant with less variability in divergence angle suggested that an enlarged meristem size promotes a shorter plastochrone, leading to an increase in the frequency of organ permutations (Landrein et al. 2015). It is likely that the permutation is not independent from the variation around the mean, and permutation may occur when the degree of variation exceeds a certain threshold (Barabé and Lacroix 2020).

### Abstract models of phyllotaxis and examination of the effect of noise

The fundamental effect of noise has been examined using the abstract model of the phyllotaxis. The model framework established by Douady and Couder (1996a, b) is based on the idea that preexisting organs inhibit the initiation of new organ primordia. Douady and Couder’s first model (Douady and Couder 1996a) assumed that the organ primordia are initiated one at a time, at a certain time interval, where the inhibitory energy exerted by preexisting primordia at the edge of the SAM, idealized as a disc, is at a minimum. The second model (Douady and Couder 1996b) discards the time-periodicity assumption and assumes one or more primordia initiate when the energy falls below a threshold value. The golden spiral patterns appeared in a wide range of parameter spaces of the models, suggesting robustness of the pattern to parameter fluctuations.

In phyllotactic pattern formation, there can be several types of noise due to different factors, such as the discrete nature of cellular tissue, inhibition strength depending on auxin level, sensitivity of cells to signals, and activity of the apex that would affect the effective radius of the generating circle and inhibitory range (Mirabet et al. 2012). Abstract models are utilized to study the effect of such noise, as the large number of parameters and details of the molecular mechanisms of realistic models may obscure the behavior of the system. At the same time, mapping between cell-based and abstract models has also been performed, enabling us to link molecular mechanisms and macroscopic behaviors of the system (Mirabet et al. 2012). Introducing noise to the abstract model reproduced some phyllotactic patterns observed in nature, for instance, a transient distichous pattern with 180° and M-shaped sequences of angles (Mirabet et al. 2012). Another model assumed that primordium initiation is a stochastic process depending on the local level of inhibition, instead of the initiation threshold employed in the abstract model (Refahi et al. 2016). Simulations of stochastic models showed dynamic behaviors not explained by the deterministic model (Refahi et al. 2016).

## Floral phyllotaxis and development

Flowers consist of several types of floral organs. Besides a few exceptions, floral organs are arranged in the order of carpels (female organs), stamens (male organs), and perianth organs, from inside to outside (Fig. 1b, c). As floral organs show spiral or whorled arrangement as leaves around a stem, it can be imagined that the organ pattern formation of floral phyllotaxis is similar to that of vegetative phyllotaxis. However, some processes in floral development are not common or not found in vegetative phyllotaxis.

### Developmental processes specific to flowers

Where and when initiation of the floral organ primordia occurs play an important role in floral development. Numerous studies on floral ontogeny have shown that it is a dynamic and diversified process. As in the development of vegetative phyllotaxis, floral organs are initiated around a floral meristem, although there are many exceptions depending on species and/or developmental stages. Some specialized organ initiations are observed, such as the ring meristem, for example in Ranunculales (Becker 2016), a common primordium that further develops into multiple organ primordia, such as in Fabaceae (Tucker 2003), and early and late fusion of primordia, such as in *Antirrhinum majus* L. (Rebocho et al. 2017).

Flowers are a determinate shoot where the number of floral organs produced is usually programmed, contrary to the indeterminate growth commonly found in vegetative shoots. The organ initiation direction along with the inside–outside axis of a flower is diversified during floral evolution. The developing vegetative apex commonly shows centripetal development, where the outer organs are initiated first and the central organ forms last. In floral development, centrifugal development, where organs develop outwards from the center, is observed in addition to centripetal development (Rudall 2010). Two types of centrifugal development are recognized: intrazonal centrifugal development, in which the organ primordia within a zone (e.g., androecium zone) show centrifugal initiation, and interzonal centrifugal development, where an entire organ zone is initiated after the zone inside it, for example when stamen initiation is earlier than petal emergence (Rudall 2010). Bidirectional prepatterning occurs where the number and position of some organs (usually stamens) are dependent on positional information related to the inner organs (carpels) (Sokoloff et al. 2018). In contrast, unidirectional prepatterning relies on positional information from preexisting organs outside the organ-generating region in centripetal development, as in vegetative shoots (Sokoloff et al. 2018). The timing of termination of the central meristem or the timing of carpel formation can affect floral organ positioning and initiation order by limiting space and/or giving positional information from inner organs.

**Fig. 3:**
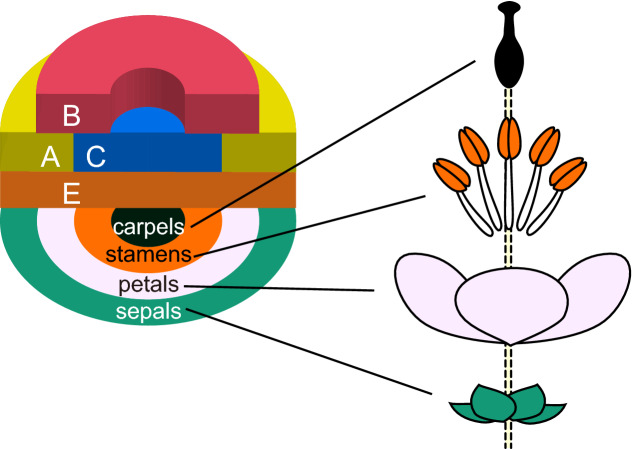
Shoot structure of a flower and ABCE model of organ-fate determination. A flower is a shoot with a very short stem (dashed lines) terminating with gynoecium formation. Lateral organs in the flower, or floral organs, are differentiated into several types depending on the expression pattern of fate determinants

Floral organ primordia acquire a specific identity depending on their position in the floral bud, and floral organ identity can change the primordium size and cause transitions between phyllotactic patterns during floral ontogeny (Wiss and Zagórska-Marek 2012). The concept of floral organ fate determination was suggested to follow an ABC model (Fig. 3), based on homeotic mutants of floral organs in two core eudicot species, *A. thaliana* and *A. majus* (Bowman et al. 1991; Coen and Meyerowitz 1991). The original model suggested concentric expression of three classes of genes (named A, B, and C), which divide the floral bud into four concentric regions and specify floral organ identity. The expression of A-class genes specifies sepal fate, A- and B-class genes specify petal fate together, B- and C-class genes specify stamen fate together, and C-class genes specify carpel fate. A later study found that another class, named E, is required for floral organ identity (Fig. 3) (Pelaz et al. 2000). The modified ABC model has been applied to other species, covering a wide range of angiosperms. In non-grass monocots such as tulips, which have two morphologically similar outer whorls, expansion of B-class gene expression to the first whorl accounts for the two petaloid perianth whorls (Kanno et al. 2007). Such shifts in the boundary of expression domain, also known as the “sliding boundary model,” can to some extent explain floral diversity (Bowman 1997; Kramer et al. 2003). Wider expressions of ABCE homologs, compared with the core eudicot model systems, are observed in other angiosperm clades such as magnoliids, Nymphaeales, Amborellales (Fig. 1a) (Chanderbali et al. 2010; Kim et al. 2005; Soltis et al. 2007; Zhang et al. 2020). The “fading borders” model has been suggested for these species, as there is a fading gradient of ABCE gene expressions observed between adjacent whorls (Buzgo et al. 2004; Soltis et al. 2007). The modified ABC model is considered to be universally applicable for organ specification in angiosperm flowers, providing a fundamental framework for understanding floral development and evolution. The differentiation itself is independent of organ positioning, but the specialized growth of each organ type can affect the initiation of subsequent organs.

Organ position can be modified by protruding position control after auxin patterning, post-meristematic regulations by boundary domain, pressure by close contact of organs, and limitation of space. The appearance of mature flowers and their development can differ, exemplified by rapid spiral initiation in calyx whorls in core eudicot species (Endress and Doyle 2007). Mathematical modelling studies have shown that the post-meristematic modification of an organ by repulsive interaction between organs enables concentric arrangement following spiral initiation and stabilizes the organ number in each circle (Kitazawa and Fujimoto 2015, 2020). This result suggests that interaction among organs, which is affected by the determinate nature of flowers and the growth control of each organ, can be crucial to ensure robust organ positioning.

### Variations in floral phyllotaxis

**Fig. 4:**
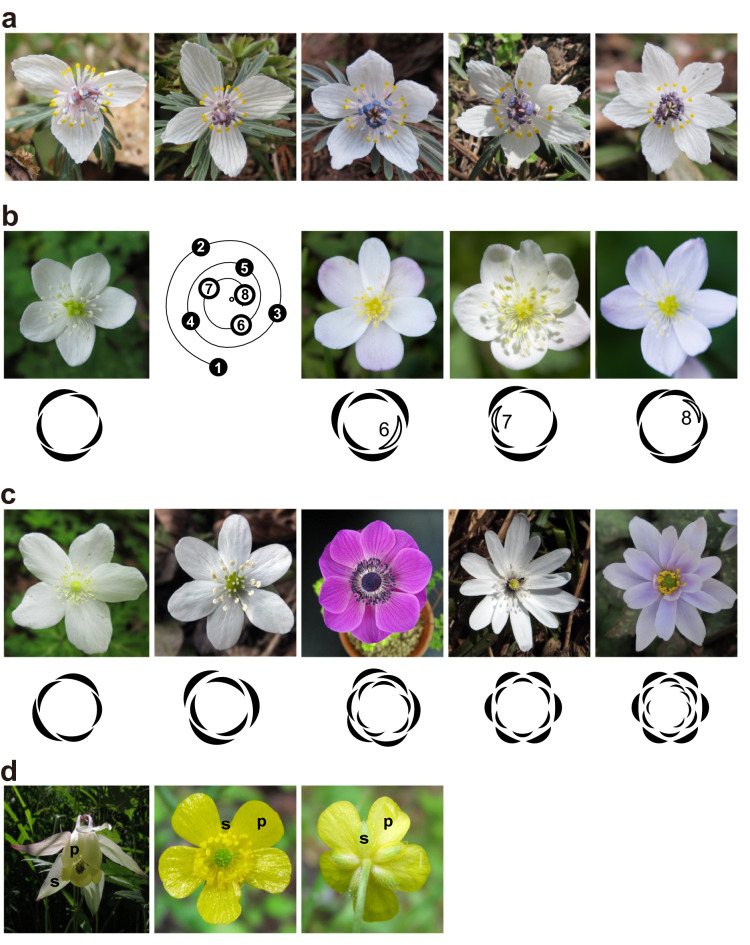
Variation and diversity in Ranunculaceae flowers. **a** Variation of sepal numbers in *Eranthis pinnatifida* Maxim. **b** Typical flower of *Anemone flaccida* F.Schmidt with five tepals (left) and flowers with six tepals with different arrangements (three panels on the right). Numbers in the schematic diagram show the initiation order of floral organs in a spiral phyllotaxis. Tepal arrangements are shown in floral diagrams below the photographs. If the sixth tepal is in the position predicted by spiral phyllotaxis, tepal arrangement must have three outer tepals and three inner tepals (middle panel). However, two other arrangements were observed, indicating that the sixth tepal is not limited to the position predicted by spiral phyllotaxis (two panels on the right). **c** *Anemone* flowers, ordered by tepal numbers. From left to right, *A. nikoensis* Maxim., *A. hepatica* var. *japonica* (Nakai) Ohwi, *A. coronaria* L., *A. raddeana* Regel, and *A. keiskeana* T.Itô ex Maxim. Floral diagrams show the arrangement of tepals in the photographs. **d** Flowers of *Aquilegia buergeriana* Siebold et Zucc. (left) and *Ranunculus* sp. (middle and right). In contrast to *Anemone* flowers, perianth organs are differentiated into sepals (s) and petals (p)

Intraspecific variations in floral traits are well-known in some species (Fig. 4a). In the nineteenth century, Hugo de Vries performed a sequence of selection of *Ranunculus bulbosus* L. (Ranunculaceae) on the petal numbers and observed changes in variation curves (de Vries 1894). Aestivation variations in *Ranunculus repens* L. was also reported in the mid-twentieth century (Cunnell 1958, 1964). Differences in organ initiation patterns among individuals have also been observed, for example, in *Magnolia* (Magnoliales) (Zagórska-Marek 1994), *Ceratophyllum* (Ceratophyllales) (Iwamoto et al. 2003), and *Anemone* (Ranunculales) (Ren et al. 2010). As in other biological processes, buffering systems for molecular stochasticity and the role of stochasticity have been suggested to influence floral development. Some features of three types of the variation, described above, are also observed in floral phyllotaxis.

Transition between regular patterns is observed in flowers, for example in *Magnolia* flowers (Wiss and Zagórska-Marek 2012; Zagórska-Marek 1994). Similar to vegetative phyllotaxis, the size ratio between floral meristems (FM) and floral organ primordia plays a central role in the determination of floral organ number and arrangement. In *Magnolia* flowers, the perianth is usually trimerous whorled and the androecium shows spiral patterns, and the relative size of organ primordia to FM size is smaller in the androecium than in the perianth (Wiss and Zagórska-Marek 2012). In *Linanthus*, flowers with a higher number of corolla lobes showed larger FM diameters (Stevens et al. 1972). Mutations in the genes required for meristem maintenance, namely *WUS* and *CLV*, show altered apical meristems not only in SAMs but also in FMs, with abnormal organ numbers and larger variations in organ positions (Clark et al. 1993, 1995; Laux et al. 1996; Schoof et al. 2000). Similar to the transient process in vegetative development, a transient deviation from normal patterns can appear between regular patterns. For example, divergence angles in early floral development oscillatory approaches 144°, equidistant angular placing of five organs in pentamerous flowers (Lyndon 1978).

Fluctuations in organ position can be caused by non-genetic factors, environmental conditions, stochastic events, and physical factors, such as external and internal pressures, and a perspective regarding flowers as dynamic random systems is being developed (Ronse De Craene 2018). Floral phyllotaxis can be altered by environmental factors, such as temperature (Crozier and Thomas 1993; Lyndon 1998). Not only the environment outside the plant body, but also the environment induced by other organs of the plant, can affect the floral development. For instance, in some mutants, the alternation of floral organ arrangements is associated with changes in the number and position of the surrounding bracts (Hepworth et al. 2005; Khan et al. 2014; Lee et al. 2007; Norberg et al. 2005). Furthermore, a correlation of floral construct with inflorescence has been observed, such as in *Adoxa moschatellina* L. with four petals in the terminal flower and five petals in the lateral flowers (Whitehead 1902). The difference between the terminal and lateral flowers can be a physical constraint by outer organs, which can change the geometry of floral meristem and lead to irregular patterns of subsequent organs (Rutishauser 2016). The expression patterns of genes can be different between the terminal and lateral flowers, depending on distinct genetic regulation between the terminal and lateral flowers (Rudall and Bateman 2003), or whether they have an inflorescence meristem to provide positional information. Such positional information, either physical or chemical, can cause differences in floral organ positioning (Nakagawa et al. 2020). A variation in floral phyllotaxis can be generated not only in the initiation of primordia but also after the initiation. For example, occasional splitting of primordia has been proposed as the mechanism underlying developmental variations in *Microseris* (Asteraceae) pappus part number, based on their Poissonian distribution (Bachmann and Chambers 1978).

In some plants with variable floral organ arrangement, exemplified by the family Ranunculaceae, the importance of organ identity determination has been emphasized as a source of variation. Floral organ primordia with different identities grow at different rates and reach different sizes; sepal primordia usually grow rapidly to cover the floral bud, in contrast to the petal primordia whose growth often occurs in the later stages of floral development. As the arrangements of previously formed organs can affect the phyllotaxis and stability of patterns of the subsequent organs (Endress 1989; Wiss and Zagórska-Marek 2012), the fluctuation in the fate determinant can cause variation in organ positioning in later developmental stages. In *Nigella* (Ranunculaceae) flowers, spiral arrangement of organ primordia has been suggested as a plausible source of the variation in organ number and arrangement (Wang et al. 2015). In spiral flowers, minor fluctuations in the expression boundary of identity determinants can be easily reflected in organ number, and this is consistent with predictions from organ number distributions (Kitazawa and Fujimoto 2014). Therefore, there is stochasticity in the expression border of organ identity genes, which can be amplified in non-whorled arrangements. Conversely, observations of mature *Anemone* flowers revealed that an occasional increase in outer organs (tepals) does not always follow the normal spiral of five tepals (Fig. 4b) (Kitazawa and Fujimoto 2016). It can be recognized as a stochastic “permutation,” as the seventh or eighth primordium acquires an outer organ fate instead of the sixth primordium. However, it is not clear whether it is a permutation in the organ initiation or fate-determination. The former possibility could be supported by variations in primordium positioning observed in Ranunculaceae species (Ren et al. 2010, 2011). The latter corresponds to a possibility that the stochasticity in the position of extra tepals may be caused by the stochastic organ fate determination depending on the expression level of fate determinants, as in stochastic initiation of organ primordia depending on inhibitory fields (Refahi et al. 2016). This could explain how variations are generated in flowers with “fading borders” of fate determinants. Although the experimental evidence for fading borders is very weak except for in the angiosperm clades that are most distant to core eudicots (Becker 2016; Voelckel et al. 2010), analogous mechanisms can exist in flowers with considerable variation in organ number and arrangement.

## Floral evo-devo and stochasticity

The number and arrangement of floral organs has played a major role in plant systematics, exemplified by Linnæus, who used stamen and carpel numbers for his taxonomy (Linnæus 1735). Although Linnæus’s taxonomy ordering plant families by stamen and carpel numbers is rejected in modern biology, it is accepted that floral organ number and arrangement are largely associated with angiosperm phylogeny. A difference in merosity, the number of organs in each whorl, is known between eudicots and the other two major clades, monocots and magnoliids (Ronse De Craene 2010; Smyth 2018). The monocot ground plan comprises two trimerous whorls of undifferentiated perianth organs, two trimerous whorls of stamens, and three fused carpels (Fig. 1b). The ground plan of magnoliids is also regarded as trimerous, but the number of whorls is higher than that of monocots (Smyth 2018). The ground plan of core eudicot (or Pentapetalae [Cantino et al. 2007]) flowers is pentamerous whorled with two perianth whorls and two stamen whorls, with the two perianth whorls morphologically differentiated into calyx (sepal whorl) and corolla (petal whorl), with alternation of organs in adjacent whorls (Fig. 1c) (Chanderbali et al. 2017; Smyth 2018). Different merosities, especially tetramery, are often found in core eudicots. In addition to these groups with relatively stable floral construction, diversity and flexibility of the floral phyllotaxis among closely related species, or within a species, is present in ‘ANA’ grade, magnoliids, and non-core eudicot clades such as Ranunculales (Damerval and Becker 2017; Endress 1990).

The function of stochasticity in floral development has been investigated using a stochastic model of the floral organ identity regulatory network (Alvarez-Buylla et al. 2008). In the model, the attractors of Boolean networks of 15 genes corresponding to four floral organ identities were approximated. Owing to the deterministic nature of the Boolean gene regulatory network, transitions between fates cannot occur. Incorporating noise into the model enabled transitioning between the attractors. Interestingly, fate transition proceeds in the order of sepal, petal, carpel, and stamen, mimicking the order of floral development in a range of noise levels (0.5–10%), and differences between the developmental timing of carpel and stamen diminish as the magnitude of noise increases. Thus, analysis of this model suggests that noise is required for cellular state transitions in floral development, and that stochasticity plays an important role, as in other biological systems.

Although stochasticity exists ubiquitously in the developmental process, the degree of noise required to change the morphology is different among species and organs. Genetic basis of robust ranges against perturbations has been evaluated in studies on the Brassicaceae species *Cardamine hirsuta* L. (Monniaux et al. 2016, 2018; Pieper et al. 2016). Most of the Brassicaceae species, including *A. thaliana*, show a stable floral construct with four sepals and four petals. On the contrary, *C. hirsuta* shows variations in petal numbers, ranging between zero and four. The variation depends on environmental perturbations such as day length and temperature, and is a heritable phenotype (McKim et al. 2017; Monniaux et al. 2016; Pieper et al. 2016). *APETALA1* (*AP1*), which establishes floral meristem identity and acts as one of the A-class genes in Brassicaceae (Litt 2007), has been shown to be involved in the difference in the petal number variation between *C. hirsuta* and *A. thaliana* (Monniaux et al. 2018). These studies suggest a genetic basis of noise buffering in Brassicaceae species with stable petal numbers, and evolutionary change in the floral stability of *C. hirsuta* by releasing cryptic variation (Monniaux et al. 2018).

It is still uncertain whether intraspecific variations in floral phyllotaxis in extant species have any function, as in molecular or cellular stochasticity. It might merely be the result of poorly buffered developmental conditions, or advantageous, as is the case in phenotypic variations in microorganisms. Studies on stochasticity in development suggest two strategies to deal with molecular or cellular stochasticity, which may or may not involve floral diversification. The first strategy is the precise regulation of organ position and gene expression, where noise is buffered out. This strategy results in a stable floral construct, and diversification occurs through further specialization and close spatial and functional association of organs, known as synorganization (Endress 1990; Endress and Doyle 2007). The second strategy is organ redundancy, which allows the maintenance of function even when the organ fate changes, as in the cellular redundancy in the vulval development of *C. elegans* (Braendle and Félix 2008). Furthermore, this can be a driving force of the floral phyllotaxis diversity among closely related species, as in Ranunculaceae (Fig. 4c, d).

Inference of the ancestral flower of extant angiosperms triggered an argument on the possible combination of floral phyllotaxis in a flower (De-Paula et al. 2018; Rümpler and Theißen 2019; Sauquet et al. 2017; Sokoloff et al. 2018). Sauquet et al. (2018) argued that the transitions of phyllotaxis in different floral organ types may be caused by gradual changes in phyllotactic parameters, such as the size ratio between meristem and organ primordium; computer models will be helpful in examining possible transitions (Douady and Couder 1996c; Zagórska-Marek and Szpak 2008). Another perspective, not considered in the approach of Sauquet et al. (2017), is the coexistence of multiple states and the possibility that the ancestral state is polymorphic (Sauquet et al. 2018). Recently, we reported the coexistence of spirals and whorls in the perianth phyllotaxis at maturity in *Anemone* and *Eranthis* (Ranunculaceae) flowers (Kitazawa and Fujimoto 2020). Studies on phyllotaxis variations suggest that such variations are common in nature, and variable phenotypes can be generated from a single developmental system, with inevitable existence of stochasticity in biological processes.

## Conclusion

Recent progress in molecular and cellular stochasticity suggested that plants utilize stochasticity in development. Although several phyllotactic patterns represented by the golden spiral appear robust, several types of variations are observed in nature, and the developmental sources of variations are discussed using mathematical models. In both vegetative and floral phyllotaxis, awareness about the importance of stochasticity has been increasing. Further clarification of the role of stochasticity in floral development will reveal new perspectives regarding the diversity of angiosperm flowers.
