# Correction to: Developmental stochasticity and variation in floral phyllotaxis

**DOI:** 10.1007/s10265-021-01307-2

**Published:** 2021-04-24

**Authors:** Miho S. Kitazawa

**Affiliations:** Center for Education in Liberal Arts and Sciences, Osaka University, 1-16 Machikaneyama-cho, Toyonaka, Osaka 560-0043 Japan

## Correction to: Journal of Plant Research 10.1007/s10265-021-01283-7

In the original publication of the article, the author name was published incorrectly as “Miho Stephanie Kitazawa”. The correct name is “Miho S. Kitazawa”.

The original article was updated.

